# *Moniezia benedeni* drives CD3^+^ T cells residence in the sheep intestinal mucosal effector sites

**DOI:** 10.3389/fvets.2024.1342169

**Published:** 2024-02-02

**Authors:** Wenzhu Chai, Wanling Yao, Jing Pan, Zhen Huang, Baoshan Wang, Bin Xu, Xiping Fan, Wanhong He, Wenhui Wang, Wangdong Zhang

**Affiliations:** ^1^College of Veterinary Medicine, Gansu Agricultural University, Lanzhou, China; ^2^Lanzhou Safari Park Management Co., Lanzhou, China

**Keywords:** *Moniezia benedeni*, sheep small intestine, CD3^+^ T cell, *laminae propria*, mucous epitheliums

## Abstract

**Introduction:**

T cells are the core of the cellular immunity and play a key role in the regulation of intestinal immune homeostasis. In order to explore the impact *Moniezia benedeni* (*M. benedeni*) infection on distributions of CD3^+^ T cells in the small intestine of the sheep.

**Methods:**

In this study, sheep pET-28a-CD3 recombinant plasmid were constructed and expressed in *BL21* receptor cells, then the rabbit anti-sheep CD3 polyclonal antibody was prepared through recombinant protein inducing. The *M. benedeni*-infected sheep (infection group, *n* = 6) and healthy sheep (control group, *n* = 6) were selected, and the distributions of CD3^+^ T cells in intestinal *laminae propria* (LP) and mucous epitheliums were observed and analyzed systematically.

**Results:**

The results showed that the rabbit anti-sheep CD3 polyclonal antibody had good potency and specificity. In the effector area of small intestine, a large number of CD3^+^ T cells were mainly diffusely distributed in the intestinal LP as well as in the mucous epitheliums, and the densities of intestinal LP from duodenum to jejunum to ileum were 6.01 cells/10^4^ μm^2^, 7.01 cells/10^4^ μm^2^ and 6.43 cells/10^4^ μm^2^, respectively. Their distribution densities in mucous epitheliums were 6.71 cells/10^4^ μm^2^, 7.93 cells/10^4^ μm^2^ and 7.21 cells/10^4^ μm^2^, respectively; in the infected group, the distributions of CD3^+^ T cells were similar to that of the control group, and the densities in each intestinal segment were all significantly increased (*p* < 0.05), meanwhile, the total densities of CD3^+^ T cells in duodenum, jejunum and ileum were increased by 33.43%, 14.50%, and 34.19%. In LP and mucous epitheliums, it was increased by 33.57% and 27.92% in duodenum; by 25.82% and 7.07% in jejunum, and by 27.07% and 19.23% in ileum, respectively.

**Discussion:**

It was suggested that *M. benedeni* infection did not change the spatial distributions of CD3^+^ T cells in the small intestine of sheep, but significantly increased their densities, which lays a foundation for further research on the regulatory mechanism of sheep intestinal mucosal immune system against *M. benedeni* infection.

## 1 Introduction

The small intestine has digestive, absorptive, secretory, and immunological functions (1, 2). Meanwhile, it is usually exposed to a variety of microorganisms [e.g., bacteria (3), viruses (4)] and parasites (5, 6), etc. The intestine can rely on multilayered defense barriers, such as mechanical-physical barriers (7), chemical barriers (8, 9) and immune barriers (9), which can prevent the invasion of pathogenic microorganisms and antigens. T cells, as the main component of lymphocytes, have biological functions such as direct killing of target cells, assisting B cells to produce antibodies, response reactions to specific antigens, and cytokine production (10). Many studies have confirmed that the toxicity of antibody dependent cell mediated cytotoxicity (ADCC) can be induced by specific antibodies that bind to the parasite. It is the key to the host’s immune response against parasitic infections (11). CD3 (cluster of differentiation 3) acts as a T cell receptor (TCR) that transduces the activation signals generated by the recognition of antigens by the TCR into T cells, resulting in T cell activation (12, 13), and is also an important surface marker molecule of T cells (14).

In intestinal mucosal immunity, *lamina propria* (LP) is an important effector site of mucosal immune responses (15). The intestinal lamina propria T cells (LPL) mainly assist B cells in synthesizing and secreting IgA (16). It can not only prevent the contact between mucosa and pathogenic microorganisms (17), neutralize and regulate the distribution of body flora (18, 19), but also play a role with complement and lysozyme, resulting in the dissolution of pathogens (20, 21), and maintain intestinal homeostasis. Intestinal intraepithelial lymphocytes (IELs) are the first immune cells in the intestinal mucosal immune system to contact foreign antigens, microorganisms, and parasites (22). It has a variety of functions, such as inhibiting mucosal hypersensitivity (23), neutralizing the cytotoxic effects of exogenous cytotoxicity (24), and secreting lymphokines (25). Most IELs contain numerous cytoplasmic granules that facilitate cytotoxic activity. Additionally, they can express effector cytokines, including interferon-gamma (IFNγ) and interleukins (IL)-2, IL-4 or IL-17. Obviously, the host can strengthen local immunity to resist infection by pathogenic microorganisms through mucosal immune-related cell proliferation (26, 27).

Parasites are a major cause of disease in livestock. According to statistics, there are 2,169 species of livestock and poultry parasites identified in China, including 404 species of nematodes, 203 species of protozoa, 373 species of trematodes, 150 species of tapeworms, 10 species of acanthocephalans and 1,030 species of arthropods (28). *Moniezia benedeni* (*M. benedeni*) is usually parasitizing the small intestines of cattle and sheep, and its main pathogenic effects are mechanical blockage, nutrient seizure, and toxicity (29). Animals exhibit weight loss as a clinical symptom, anemia, localized gastrointestinal distension, dysentery or severe constipation (30), and common psychiatric symptoms such as spasms, gyratory movements, head tilting and empty chewing, causing death in some severe cases (31). When the parasite invades the host, it can trigger the host’s immune response (32), mainly type II immune responses, which involves the production of specific immunoglobulins and cytokines, promoting the proliferation of intestinal epithelial cells and the increase of intestinal mucus to promote the excretion of the parasite (33). The results showed that in *cysticular echinococcus* infection, the expression of host’s CD69, CD44 and CD40L of CD4^+^ and CD8^+^ T cells was up-regulated and the expression of CD62L was down-regulated. The number of regulatory T cells expressing CD4^+^ CD25^+^ FoxP3^+^ increased significantly (34). Our previous studies have confirmed that *M. benedeni* infection significantly reduces the density of small intestinal IgA^+^, IgG^+^, and IgM^+^ cell distributions (35). However, the effect of *M. benedeni* infection on the distribution and expression of T-lymphocytes in the effector sites in the small intestine in sheep and the resulting anti-parasitic immune response are not clear. The aim of this study is to analyse the distribution characteristics and distribution density of CD3^+^ T cells in the effector zone of the small intestine of sheep infected with the cestode *M. benedeni* through bioinformatic analysis of CD3, preparation of polyclonal antibodies, immunohistochemistry and immunofluorescence. On this basis, the study of the effect of *M. benedeni* infection on the pattern of changes in T lymphocytes in sheep’s small intestine lays the foundation for further elucidating the regulatory mechanism of the sheep intestinal mucosal immune system in response to *M. benedeni* infection.

## 2 Materials and methods

### 2.1 Experimental animals and experimental design

Uninfected (control group, *n* = 6) and *M. benedeni* infected sheep (infected group, *n* = 6) were selected, respectively. They were anaesthetised intravenously with sodium pentobarbital (20 mg/kg) and then exsanguinated to death. Secondly, the abdominal cavity of the executed sheep was opened, and the duodenum, jejunum and ileum tissues were quickly cut out, and the tissue samples taken were fixed in 4% formaldehyde solution, embedded and sliced according to the conventional methods to make paraffin sections (4 μm). All tissue samples of the duodenum, jejunum and ileum were collected in sterile tubes for ELISA and western blotting detection. The histological samples of them were fixed in a 4% neutral paraformaldehyde solution for more than 15 days. Purchase of healthy male New Zealand White rabbits from the Laboratory Animal Center of Lanzhou Institute of Veterinary Medicine, Chinese Academy of Agricultural Sciences, China, weight about 2.2 kg.

### 2.2 Preparation and western blotting analysis of polyclonal antibody against CD3 in sheep

Referring to the coding region (CDS) of the sheep CD3 gene sequence (GenBank: S53077.1), the mRNA has 1,343 bp in length, with a coding region from position 134 to 713, and it translates into a protein consisting of 193 amino acids. The prediction of transmembrane structure revealed a total of 115 amino acids for CD3 in the extramembrane region, and this extramembrane portion (1–114) was intercepted using Editseq (DNAStar 7.0). The signal peptide was predicted and truncated (1–21), leaving 95 amino acids, which correspond to a base sequence of 285. Then the enzyme cutting site was determined, and finally sent to Genewiz Biotechnology Co., Ltd. for sequence synthesis. The CD3 was connected with pET-28a (+) vector, and transformed into DH5α receptor cells. Finally, the correctly sequenced positive recombinant plasmid was obtained as pET-28a-CD3.

The constructed pET-28a-CD3 recombinant plasmid was transfected into 50 μL of *BL21*(DE3) competent cells under aseptic conditions. Single colony were picked in 5 mL of LB liquid medium containing Kan^+^, then cultured at 37°C and 220 rpm on a shaker overnight. The overnight bacteria were transferred into 5 mL of LB liquid medium containing Kan^+^ according to 1:100, cultured at 37°C and 220 rpm until the OD_600_ value reached 0.6–0.8. One milliliter fluid was taken in a 1.5 mL centrifuge tube as the preinduction control, and the remaining solution was added with 1 mol/L IPTG according to 1:1000. The cultured was induced at 37°C and 220 rpm for 6 h. One milliliter fluid was taken in a 1.5 mL centrifuge tube for the post-induction control. The precipitate was collected by ultrasonically crushing in an ice bath, and the supernatant and precipitate were separately collected and sampled for SDS-PAGE. The collected precipitates were combined with affinity column (containing HIS-tagged proteins), and the proteins were purified. The concentration of the proteins was determined spectrophotometrically. Purified sheep CD3 recombinant protein was emulsified and injected at multiple points into rabbits, targeting the popliteal lymph nodes and subcutaneously on the back. After four immunizations, blood was collected from the heart and centrifuged to obtain rabbit anti-sheep CD3 polyclonal antibodies.

The purified recombinant protein was electrophoresed on a 15% SDS-PAGE and transferred to PVDF membrane, which was sealed by adding skimmed milk powder at 37°C. The PVDF membrane was conjugated with rabbit antiserum (diluted 1:500) and incubated overnight at 4°C. After being washed 3 times with TBS-T, the HRP-labeled secondary antibody (diluted 1:8000) was added and incubated for 2 h at room temperature. Finally, after being washed 3 times with TBS-T, the ECL luminescent solution was added for color development.

### 2.3 Immunohistochemical staining procedures

The paraffin sections were dewaxed with water, placed in citrate buffer (power 900 W, action 10 min), cooled naturally, and washed with distilled water for 2 min × 3 times. Then, they were treated with 3% hydrogen peroxide at room temperature for 15 min and washed again with distilled water for 2 min × 3 times; the distilled water surrounding the tissues was drained off using filter paper. Then 5% BSA (from a ready-to-use immunohistochemical staining kit) blocking solution was added dropwise and allowed to act at 37°C for 40 min. The excess liquid was shaken off from the tissues, and diluted primary antibody was added dropwise. The tissues were incubated at 4°C overnight. After washing with PBS (0.01 mol/L, pH 7.2) for 2 min × 3 times, the secondary antibody (goat anti-mouse/rabbit IgG of HRP) was added dropwise and incubated at 37°C for 30 min. After washed with PBS for 5 min × 4 times, the appropriate amount of SABC was added dropwise, incubated at 37°C for 30 min, and washed with PBS for 5 min × 4 times; DAB color development kit (20×, Goods number: ZLI-9018, Beijing Zhongsui Jinqiao Biotechnology Co., Beijing, China) was used at room temperature, and the reaction was terminated by washing with water. Then the nucleus were stained with hematoxylin for 50 s, washed with water for 10 min, differentiated for 5 times. Dehydrated, and mounted with neutral balsam. Serial sections were made, and the antibody stock solution was diluted to 1:200, 1:400, 1:600, 1:800, 1:1000, 1:1200, respectively, to observe the staining effect, and the concentration was finally determined to be 1:600.

### 2.4 Statistical analysis

Using a digital scanner (3DHISTECH Pathology Slice Scanner, Shandong Spirit Medical Technology Co.) to observe the location and characteristics of CD3^+^ T cell distribution. For each segment of intestine, 5 slices were selected and 10 visual field of mucous epitheliums were randomly selected for each slice; the number of positive cells in each mucous epithelium was counted, and the density of positive cells was calculated. Statistical analysis was performed using Origin 2022 and SPSS 23.0 software, using one-way ANOVA (LSD method was used for *post hoc* analysis) to analyze the differences between the distribution densities of positive cells between the groups; and the *t*-test of independence was used to analyze the significance of the differences between the groups infected with the same site and the control group, the significant difference was considered at *p* < 0.05.

### 2.5 Immunofluorescence staining

Paraffin sections were dewaxed to water, placed in citrate buffer (power 900 W, action 10 min), cooled naturally, and washed in distilled water for 2 min × 3 times. Shake off the liquid around the section, draw a circle around the tissue with a histochemical pen (to prevent the antibody from flowing out), and incubate the circle with a drop of BSA for 30 min. Gently shake off the sealing solution, dilute the sections with primary antibody (1:600), and incubate the sections in a wet box at 4°C overnight. The slides were washed in PBS on a shaker for 5 min × 4 times, and the secondary antibody (Goat Anti-Rabbit IgG H&L (Alexa Fluor^®^ 488) ab150077, Abcam) was added dropwise for 50 min at room temperature and protected from light. The slides were placed in PBS and washed on a shaking table for 5 min × 4 times, and DAPI staining solution was added dropwise in the circle, and incubated at room temperature and protected from light for 10 min. The slides were placed in PBS and washed on a shaking table for 5 min × 4 times. Spontaneous fluorescence quencher was added to the circle for 5 min, and rinsed under running water for 10 min. The sections were shaken dry and sealed with an anti-fluorescence quenching sealer. Finally, the distribution of CD3^+^ T cells in sheep small intestine was observed under a fluorescence microscope, and the images were acquired (The DV Elite^™^ Imaging System, GE, United States; DAPI UV excitation wavelength 330–380 nm, emission wavelength 420 nm, blue light; FITC excitation wavelength 465–495 nm, emission wavelength 515–555 nm, green light).

## 3 Results

### 3.1 Similarity comparison and phylogenetic tree construction of CD3 in sheep

**Figure 1:**
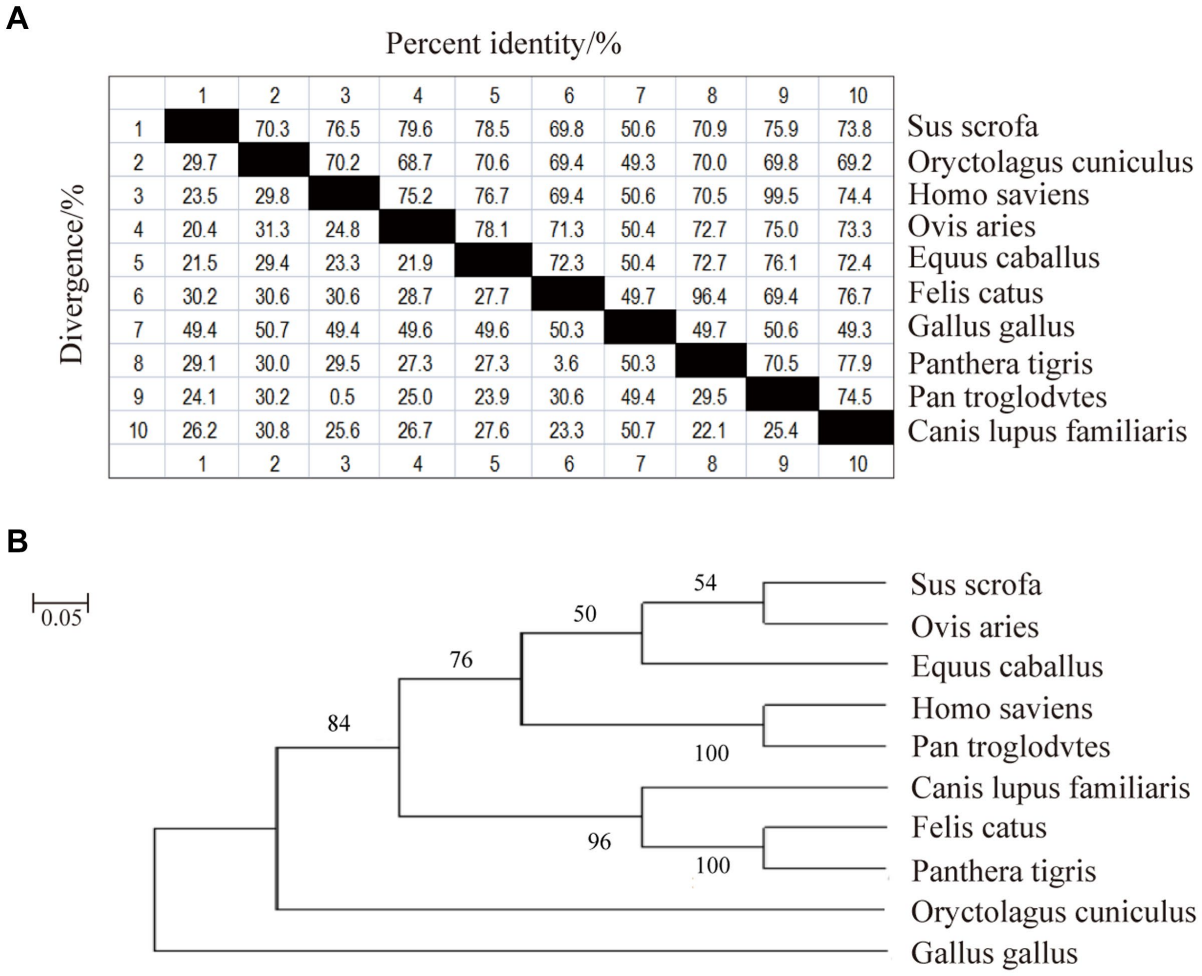
Nucleotide sequence similarity analysis and phylogenetic tree of sheep CD3 gene. **(A)** CD3 nucleotide sequence similarity analysis. **(B)** CD3 nucleotide sequence evolutionary tree analysis.

Phylogenetic homology comparisons and phylogenetic tree construction were performed using MAGA11.0 software based on the CDS region of the CD3 gene sequences obtained from the NCBI database for pig, rabbit, human, sheep, horse, domestic cat, chicken, tiger, chimpanzee and dog. As shown in Figure 1A, the similarity between pig and rabbit, human, sheep, horse, domestic cat, chicken, tiger, chimpanzee and dog CDS regions was 70.3%, 76.5%, 79.6%, 78.5%, 69.8%, 50.6%, 70.9%, 75.9%, and 73.8%, respectively. Sheep and pigs were found to be the most closely related species (Figure 1B). They were followed by humans, chimpanzees, domestic cats and tigers. In contrast, chicken were found to be the most distantly related species (Figure 1).

### 3.2 Bioinformatics analysis of CD3 in sheep

#### 3.2.1 Physical and chemical properties

CD3 encodes 192 amino acids with a molecular weight of 21555.57U; the theoretical isoelectric point (PI) is 6.73, indicating that CD3 is an acidic protein; and the predicted instability index (PI) is 19.83, proving that CD3 is a stable protein. According to Table 1, leucine was the most abundant among the 20 amino acids encoding CD3, accounting for 22%, or 11.5%, while phenylalanine was the least abundant with only 2%, or 1.0% (Table 1). The predicted theoretical half-lives were 30 h in mammalian reticulocytes cultured *in vitro*, > 20 h in yeast, and >10 h in *E. coli*.

**Table 1:** Amino acid composition of CD3 in sheep.

Amino acids	Quantity	Proportion	Amino acids	Quantity	Proportion
Ala(A)	11	5.7%	Thr(T)	10	5.2%
Cys(C)	6	3.1%	Gly(G)	17	8.9%
Arg(R)	11	5.7%	Asn(N)	13	6.8%
Asp(D)	7	3.6%	Gln(Q)	11	5.7%
Glu(E)	14	7.3%	His(H)	2	1.0%
Ile(I)	6	3.1%	Leu(L)	22	11.5%
Lys(K)	10	5.2%	Met(M)	5	2.6%
Phe(F)	2	1.0%	Pro(P)	10	5.2%
Ser(S)	9	4.7%	Trp(W)	4	2.1%
Tyr(Y)	9	4.7%	Val(V)	13	6.8%

#### 3.2.2 Prediction of hydrophilic/hydrophobicity, transmembrane regions and signaling peptides

**Figure 2:**
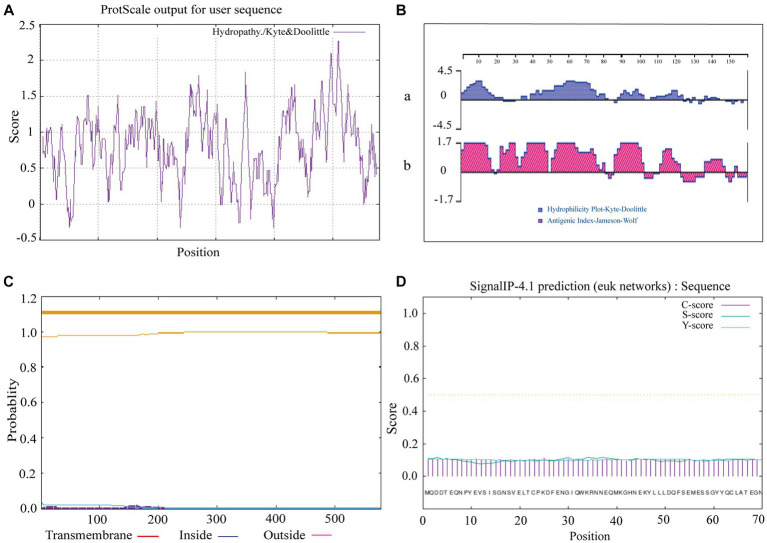
Ovine CD3 hydrophilicity, antigenic epitopes, protein transmembrane regions and signal peptide prediction **(A)**. Prediction of hydrophobicity of sheep CD3 protein. **(B)** Prediction of hydrophilicity and antigenic epitope of sheep CD3 [**(a)** hydrophilicity prediction; **(b)** antigenic epitope prediction]. **(C)** Prediction of transmembrane region of sheep CD3 protein (red line, transmembrane region; blue line, intramembrane region; purple line, extra-membrane region). **(D)** Sheep CD3 signal peptide prediction (C-score, shear site value; S-score, signal peptide region value; Y-score, parameter combining S- and C-values).

The prediction of hydrophobicity results for the amino acid sequence of sheep CD3 protein (Figure 2A), revealed that the lowest value was at amino acid position 50/240/399, which was −0.322 and the most hydrophilic. The highest value is at amino acid position 508/509, which was 2.267 and the most hydrophobic. Amino acids in the hydrophilic region accounted for more amino acids than those in the hydrophobic region, so the sheep CD3 protein was a hydrophilic protein. The prediction analysis of hydrophilicity (Figure 2Ba) and antigenic epitope (Figure 2Bb) indicated that it was a hydrophilic protein with high antigenic index. The transmembrane structure predicted that all the amino acids were absent from the transmembrane region (Figure 2C). The signal peptide predicted that the protein did not have a signal peptide structure (Figure 2D).

#### 3.2.3 Secondary structure projections and three-tier structural projections

**Figure 3:**
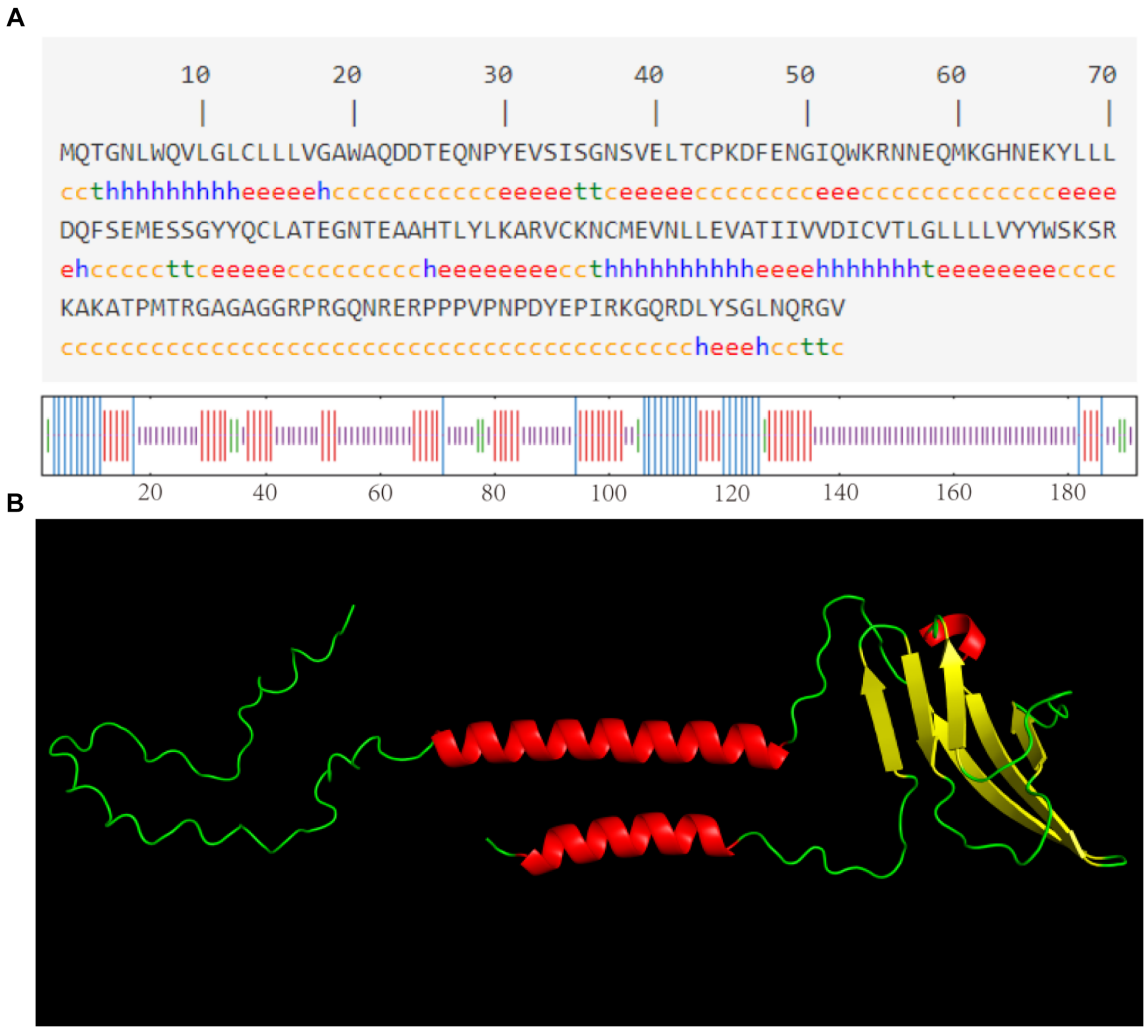
**(A)** Predicted secondary structure of sheep CD3 protein h, α-helix; e, extended chain; t, β-turn; c, irregularly coiled. **(B)** Predicted tertiary structure of CD3 protein.

As shown in Figure 3A, the CD3 protein consists of α-helix (16.15%), extended strand (26.56%), β-turn (4.69%), and irregular curl (52.60%) regions, with the highest percentage being accounted for by the CD3 protein’s irregular curl region. This suggests that there are larger binding sites within CD3, indicating its classification as a mixed-type protein with a more complex biological function. The tertiary structure of sheep CD3 protein was predicted using the online sequencing software SWISS-MODEL,^1^ and the coverage of the prediction model was 98%, which indicated that the model was reasonably constructed. The results of the tertiary structure model prediction showed the consistency with the results of the secondary structure prediction (Figure 3B).

#### 3.2.4 Analysis of phosphorylation and glycosylation sites and protein interactions of CD3 in sheep

**Figure 4:**
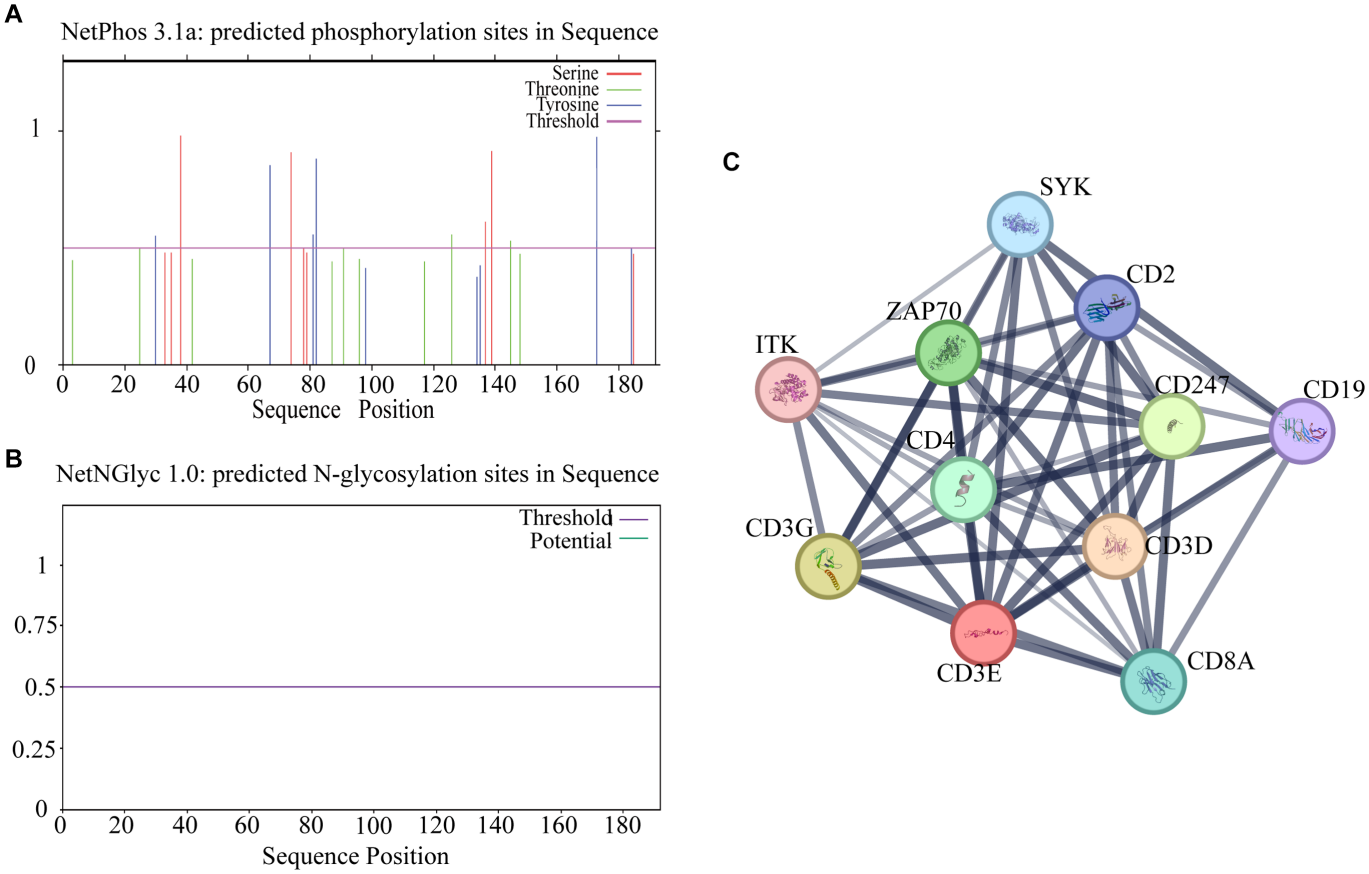
Analysis of potential phosphorylation and glycosylation sites of CD3 in sheep and other protein interactions. **(A)** Phosphorylation site prediction. **(B)** Glycosylation site prediction. **(C)** Protein interaction analysis. The thickness of the lines indicates the intensity of the interaction between proteins, and the thicker the lines indicate the greater the interaction between proteins.

Online software for its phosphorylation^2^ and glycosylation^3^ prediction analysis revealed 19 specific phosphorylation sites and no glycosylation sites (Figures 4A,B). STRING analysis showed an average local clustering coefficient of 0.941. As shown in Figure 4C, there are interactions between sheep CD3 and proteins such as CD28, ITK, CD3G, CD3D, SYK, CD4, CD247, ZAP70, LCK, CD19, and CD8A; these proteins exhibit strong interconnections among themselves.

### 3.3 Preparation for anti-sheep CD3 polyclonal antibodies

**Figure 5:**
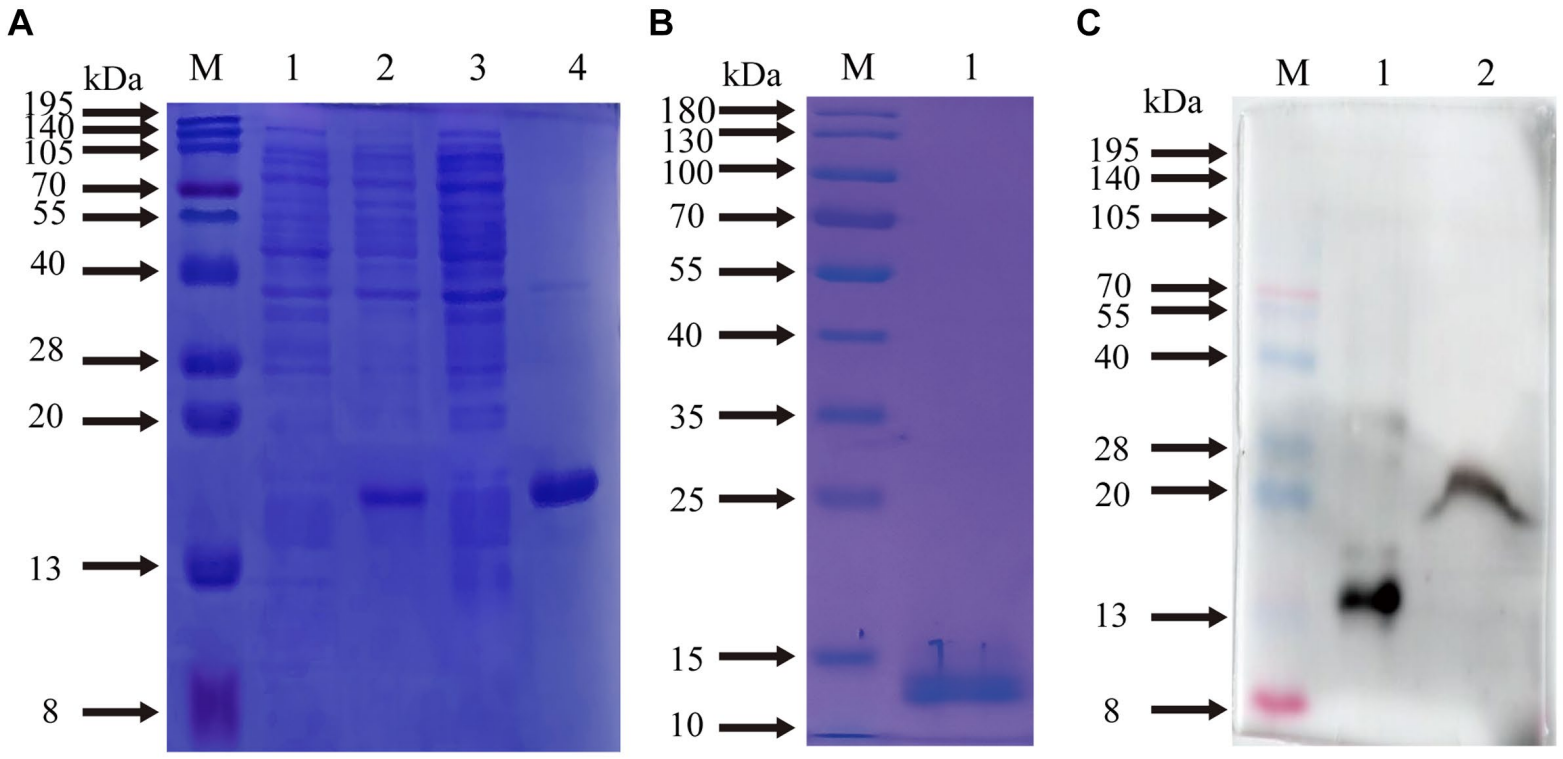
Prediction of CD3 expression form in sheep and WB results. **(A)** M. protein molecular quality standard; (1) recombinant bacterial pre-induction product; (2) recombinant bacterial post-induction product; (3) supernatant of recombinant bacterial induction product; (4) precipitation of recombinant bacterial induction product. **(B)** M. protein molecular quality standard; (1) purified proteins. **(C)** M. protein molecular quality standard; (1) purified proteins; (2) natural total proteins.

**Figure 6:**
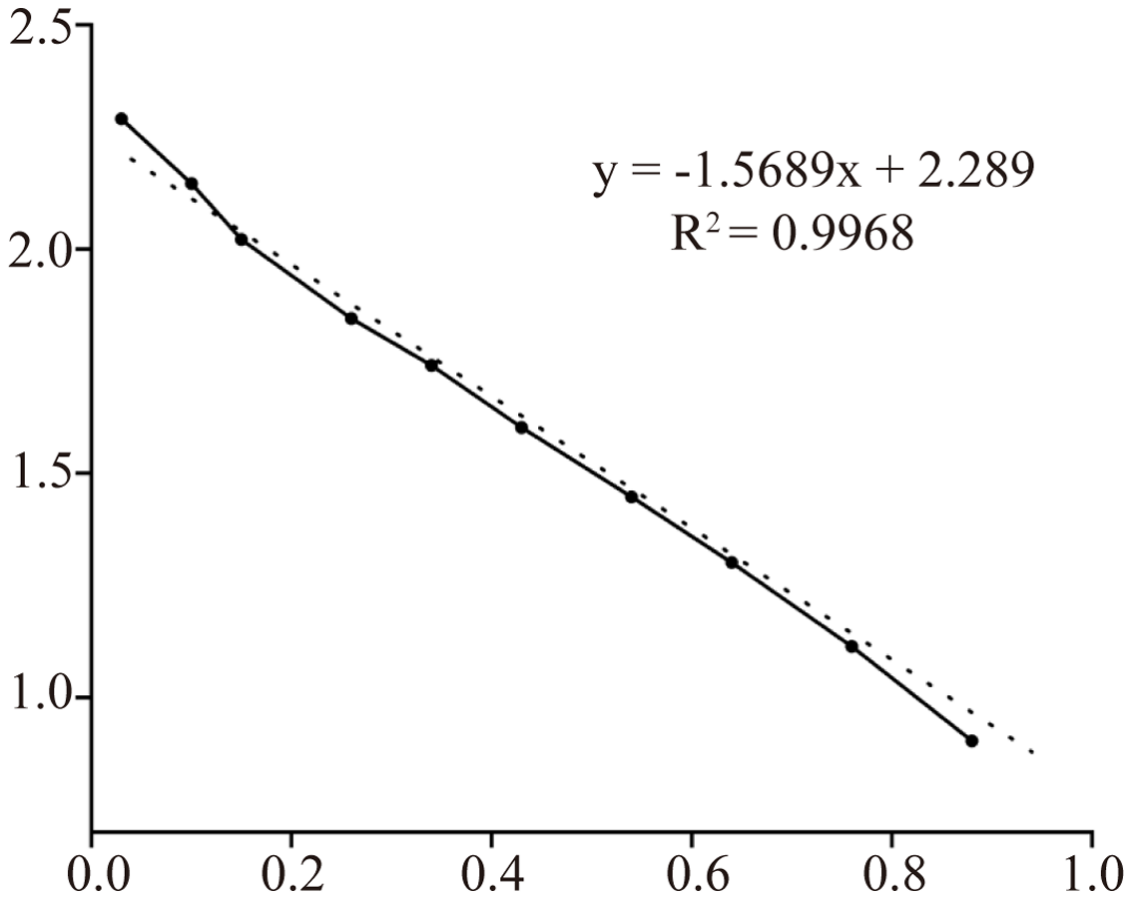
Protein relative molecular mass standard curve.

The supernatants and precipitates of the sonicated proteins were collected separately for SDS-PAGE. The results showed that compared with the products without induction by recombinant bacteria, the post-induction products appeared obvious expression bands (Figure 5A). Additionally, target bands appeared in the precipitates of the recombinant bacteria-induced products after sonication and centrifugation. It indicates that the recombinant protein CD3 is successfully expressed in *BL21* and exists as an inclusion body. The standard curve was obtained by plotting the relative mobility against the logarithm of the molecular weight of standard proteins (Figure 6). After elution, the purified recombinant protein was found to be free of heterogeneous proteins as detected by SDS-PAGE (Figure 5B), which indicated that the protein was of high purity. The results of western blotting showed that there was a clear protein blot appearing at about 13.7 kDa on the PVDF membrane (Figure 5C), which indicated that the rabbit anti-sheep CD3 antibody could specifically bind to the recombinant protein.

Based on the relative mobility of the protein to be measured, its relative molecular mass was determined from the standard curve (Table 2). The correlation coefficient *R*^2^ > 0.99 indicates that the established standard curve can be used to determine the relative molecular mass of the protein. The mobility of CD3 in electrophoresis was found to be 3.69, and the calculated relative molecular mass of CD3 is 13.7 kDa.

**Table 2:** Determination of protein band mobility in CD3-SDS-PAGE.

Enterprise	Measured value									
Relative molecular mass M/kDa	195	140	105	70	55	40	28	20	13	8
Logarithm of relative molecular mass LgM	2.29	2.14	2.02	1.84	1.74	1.60	1.44	1.30	1.11	0.90
Sample migration distance L/cm	0.15	0.50	0.75	1.30	1.70	2.15	2.70	3.20	3.80	4.40
Relative mobility Mr	0.03	0.1	0.15	0.26	0.34	0.43	0.54	0.64	0.76	0.88

### 3.4 The pattern of the effect of *Moniezia benedeni* infection on the distribution of CD3^+^ T cells

**Figure 7:**
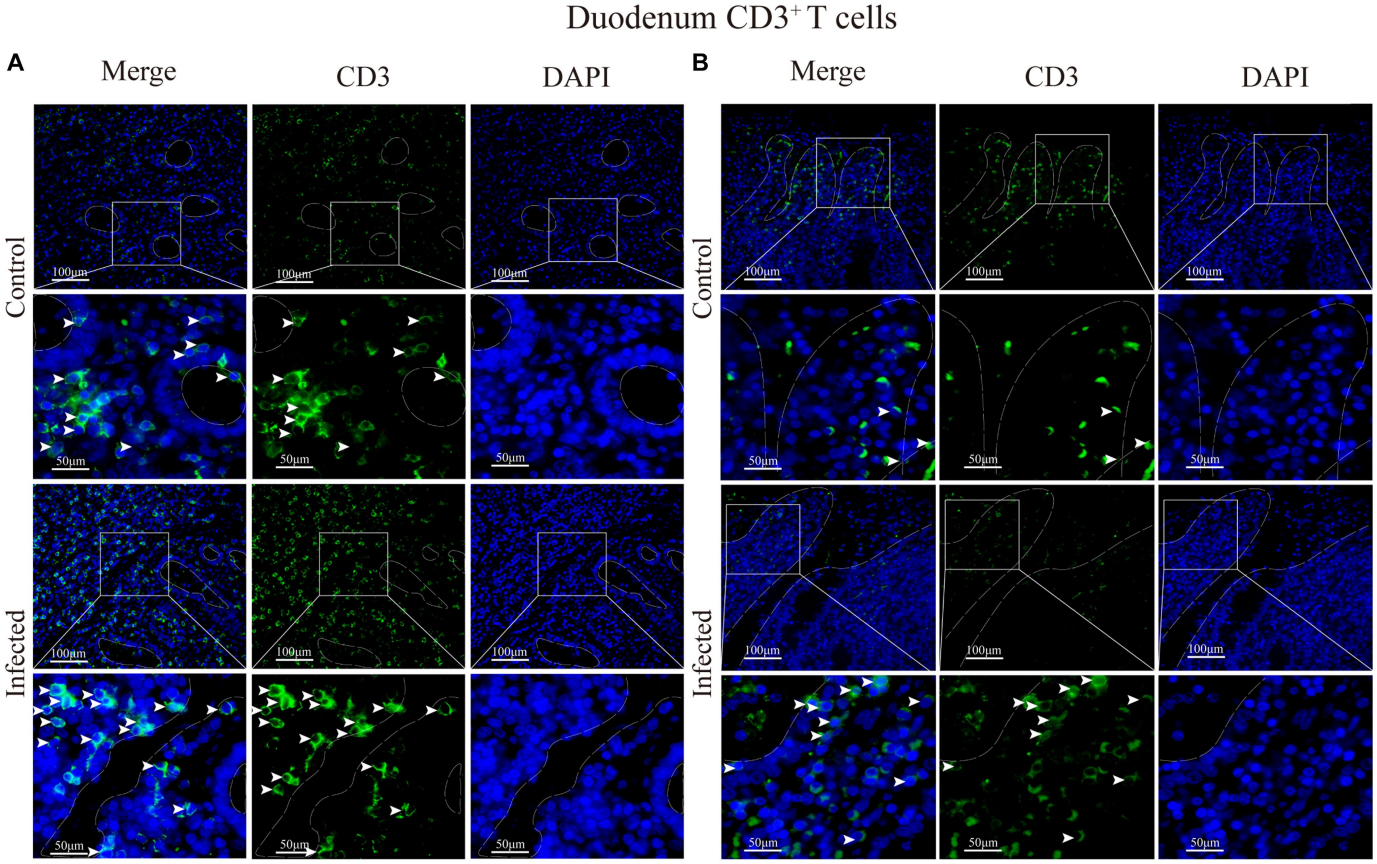
**(A)** Distribution of CD3^+^ T cells in the intestinal LP of the duodenum. **(B)** Distribution of CD3^+^ T cells in the duodenal mucous epitheliums. The white arrows represent T cells.

**Figure 8:**
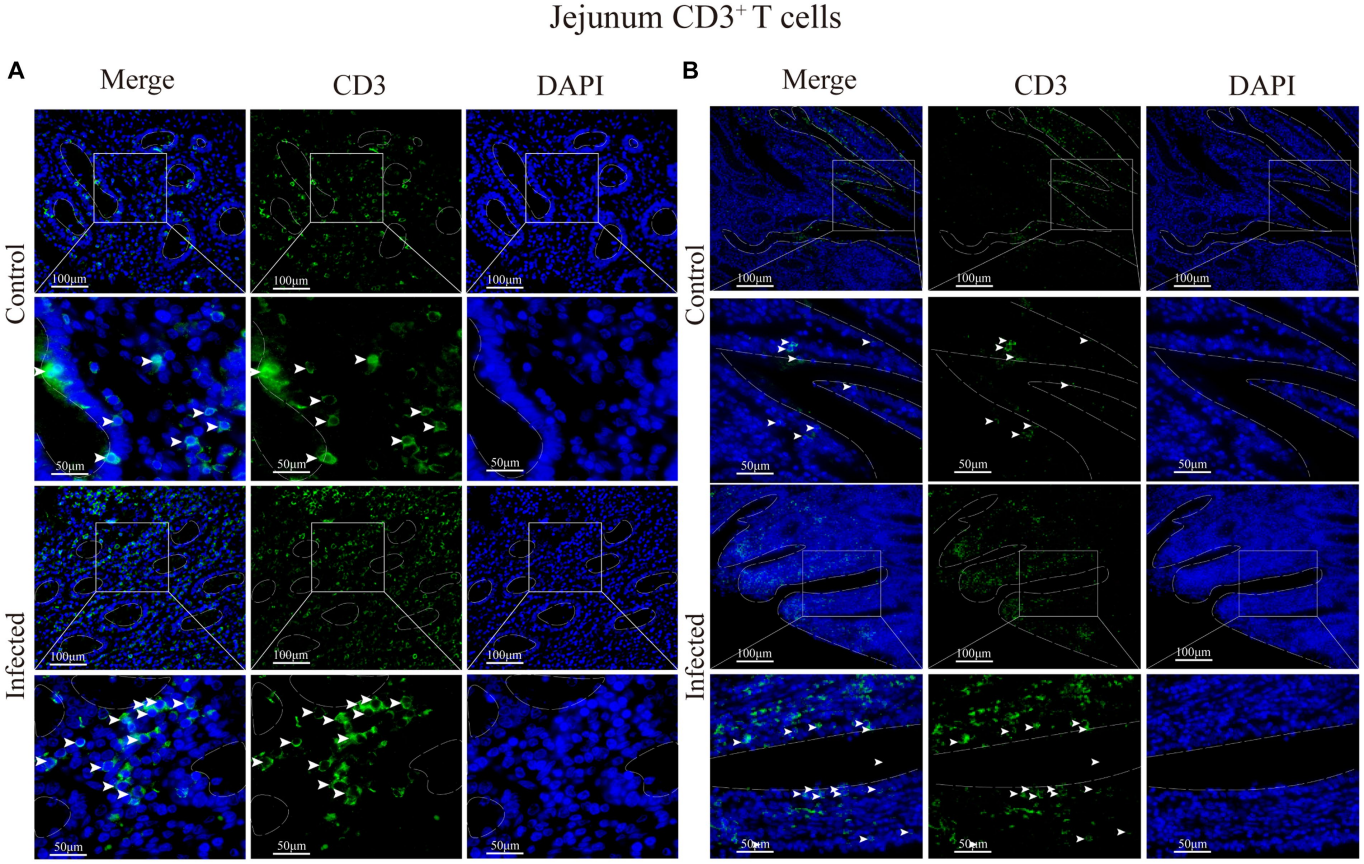
**(A)** Distribution of CD3^+^ T cells in the intestinal LP of the jejunum. **(B)** Distribution of CD3^+^ T cells in the duodenal mucous epitheliums. The white arrows represent T cells.

**Figure 9:**
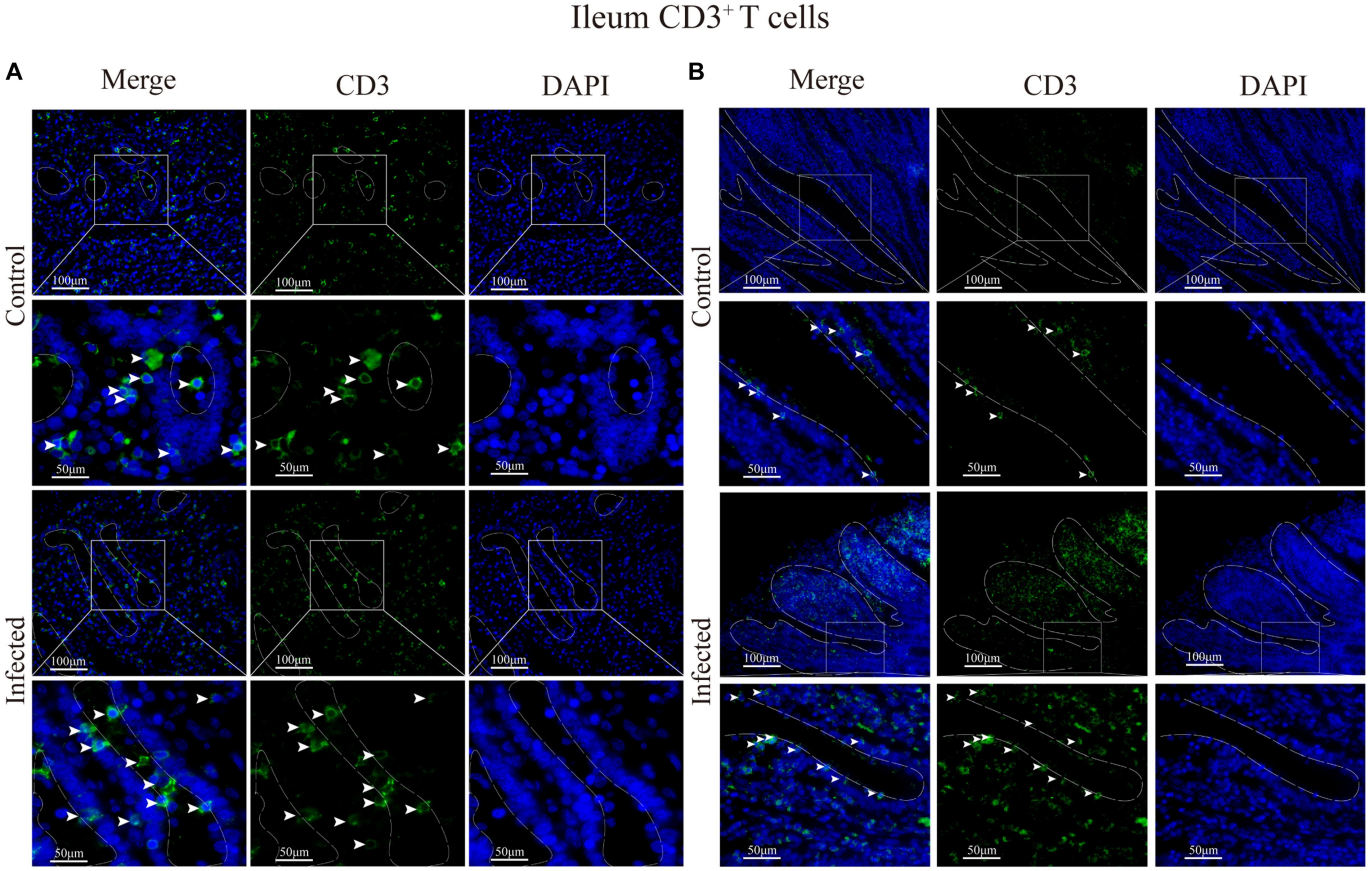
**(A)** Distribution of CD3^+^ T cells in the intestinal LP of the ileum. **(B)** Distribution of CD3^+^ T cells in the ileal mucous epitheliums. The white arrows represent T cells.

**Figure 10:**
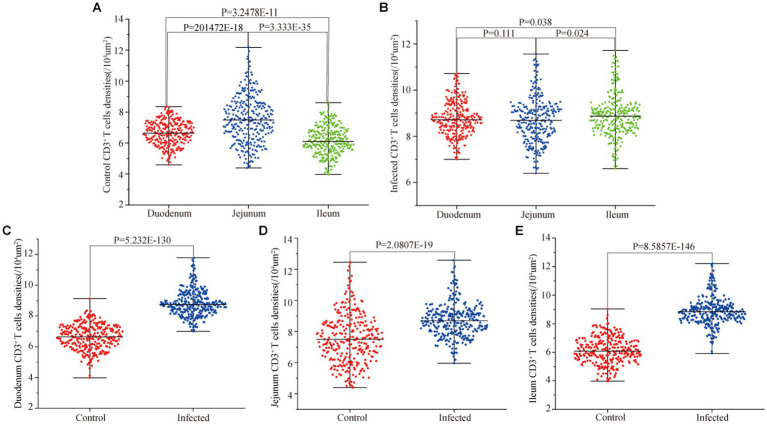
Effect of *M. benedeni* infection on the distribution density of small intestinal CD3^+^ T cells in sheep. **(A)** Distribution density of small intestinal CD3^+^ T cells in control sheep. **(B)** Distribution density of small intestinal CD3^+^ T cells in infected sheep. **(C)** Distribution density of duodenal CD3^+^ T cells by *M. benedeni* infection. **(D)** Distribution density of jejunum CD3^+^ T cells by *M. benedeni* infection. **(E)** Distribution density of small intestinal CD3^+^ T cells by *M. benedeni* infection on ileal CD3^+^ T cell distribution density; *p* < 0.05 indicates significant difference.

Immunofluorescence results showed that sheep CD3^+^ T cells were mainly diffusely distributed around the intestinal LP and within the mucous epitheliums of the duodenum (Figures 7A,B), jejunum (Figures 8A,B) and ileum (Figures 9A,B). Immunohistochemical results showed that the distribution densities of CD3^+^ T cells in each intestinal segment, from the duodenum to the jejunum and ileum were 6.64 cells/10^4^ μm^2^, 7.62 cells/10^4^ μm^2^ and 6.15 cells/10^4^ μm^2^, respectively. The highest distribution density was found in the jejunum, followed by the duodenum and ileum. After *M. benedeni* infection, the distribution densities of total CD3^+^ T cell were significantly increased (Table 3 and Figure 10), with densities of 8.86 cells/10^4^ μm^2^ (duodenum), 8.73 cells/10^4^ μm^2^ (jejunum) and 8.93 cells/10^4^ μm^2^ (ileum), respectively. Each density increased by 33.43% (duodenum), 14.50% (jejunum) and 34.19% (ileum).

**Table 3:** Changes in the density of CD3^+^ T cells in the small intestine of sheep after *M. benedeni* infection.

	Duodenum	Jejunum	Ileum
Control (cells/10^4^ μm^2^)	6.64 ± 0.83^Aa^	7.62 ± 1.68^Ba^	6.15 ± 0.92^Ca^
Infected (cells/10^4^ μm^2^)	8.86 ± 0.88^Ab^	8.73 ± 1.17^Ab^	8.93 ± 1.02^Bb^
Rate of increase	33.43%	14.50%	34.19%

Rise rate = (infected group − control group)/control group×100%. Differences in data in the same row are indicated by capital letters, and different letters indicate significant differences (*p* < 0.05); differences in data in the same column are indicated by lowercase letters, and different letters indicate significant differences (*p* < 0.05).

**Figure 11:**
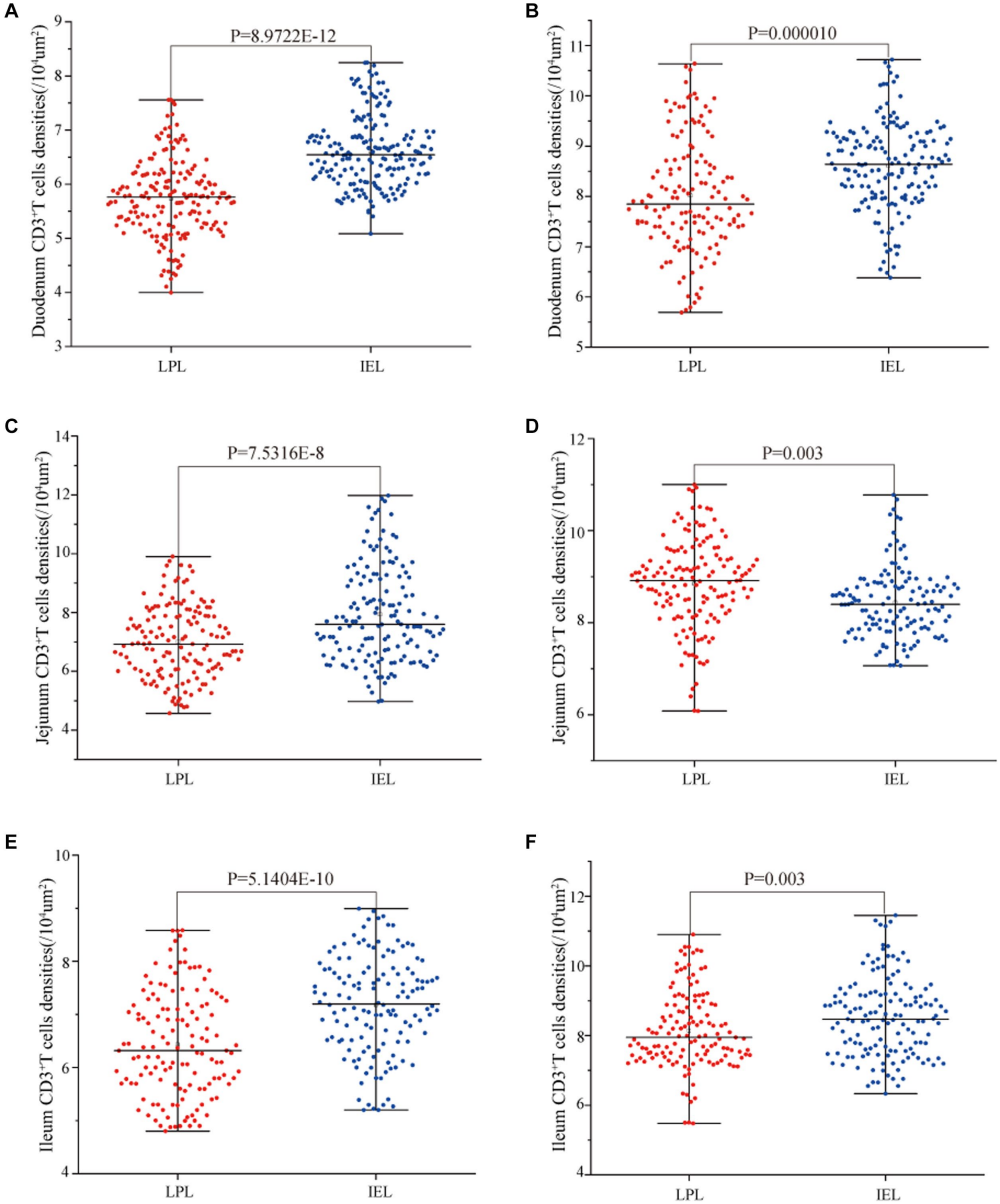
Effect of *M. benedeni* infection on the distribution of CD3^+^ T cells in the intestinal LP and mucous epitheliums in the small intestine of sheep **(A,B)** are the distribution densities of CD3^+^ T cells in the intestinal LP and mucous epitheliums of the duodenum by *M. benedeni* infection; **(C,D)** are the distribution densities of CD3^+^ T cells in the intestinal LP and mucous epitheliums of the jejunum by *M. benedeni* infection; **(E,F)** are the distribution densities of CD3^+^ T cells in the intestinal *LPxaq* and mucous epitheliums of the ileum by *M. benedeni* infection. Distribution density of CD3^+^ T cells (**A,C,E** are control groups, and **B,D,F** are infection groups). *p* < 0.05 indicates significant difference.

Statistical analysis of the distribution density of CD3^+^ T cells within mucous epitheliums and intestinal LP in each intestinal segment showed that the distribution densities of CD3^+^ T cells on the mucous epitheliums were 6.71 cells/10^4^ μm^2^ (duodenum), 7.93 cells/10^4^ μm^2^ (jejunum) and 7.21 cells/10^4^ μm^2^ (ileum), respectively. The distribution densities of CD3^+^ T cells on the intestinal LP were 6.01 cells/10^4^ μm^2^ (duodenum), 7.01 cells/10^4^ μm^2^ (jejunum) and 6.43 cells/10^4^ μm^2^ (ileum), respectively. The distribution densities of CD3^+^ T cells in the mucous epitheliums of each intestinal segment after *M. benedeni* infection were 8.59 cells/10^4^ μm^2^ (duodenum), 8.49 cells/10^4^ μm^2^ (jejunum) and 8.60 cells/10^4^ μm^2^ (ileum), respectively, with an increase of 27.92% (duodenum), 7.07% (jejunum) and 19.23% (ileum) in the intestinal LP of each intestinal segment. The distribution densities of CD3^+^ T cells were 8.03 cells/10^4^ μm^2^ (duodenum), 8.82 cells/10^4^ μm^2^ (jejunum) and 8.17 cells/10^4^ μm^2^ (ileum), which increased by 33.57% (duodenum), 25.82% (jejunum) and 27.07% (ileum), respectively (Table 4 and Figure 11). The most dramatic increase in the distribution density of CD3^+^ T cells in jejunal mucous epitheliums and intestinal LP was observed (*p* < 0.05).

**Table 4:** Changes in the density of CD3^+^ T cells in the mucous epitheliums and the intestinal LP of sheep small intestine after *M. benedeni* infection.

		Duodenum	Jejunum	Ileum
Mucous epitheliums	Control (cells/10^4^ μm^2^)	6.71 ± 0.88^Aa^	7.93 ± 1.01^Ba^	7.21 ± 1.01^Ca^
	Infected (cells/10^4^ μm^2^)	8.59 ± 0.99^Ab^	8.49 ± 0.88^Ab^	8.60 ± 0.80^Bb^
	Rate of increase	27.92%	7.07%	19.23%
Intestinal LP	Control (cells/10^4^ μm^2^)	6.01 ± 0.82^Aa^	7.01 ± 0.73^Ba^	6.43 ± 1.01^Ca^
	Infected (cells/10^4^ μm^2^)	8.03 ± 1.01^Ab^	8.82 ± 0.83^Bb^	8.17 ± 0.77^Cb^
	Rate of increase	33.57%	25.82%	27.07%

Rise rate = (infected group − control group)/control group × 100%. Differences in data in the same row are indicated by capital letters, and different letters indicate significant differences (*p* < 0.05); differences in data in the same column are indicated by lowercase letters, and different letters indicate significant differences (*p* < 0.05).

## 4 Discussion

The results showed that sheep CD3 consisted of 192 amino acids, which had a high affinity with pigs (about 79.6%) and the lowest affinity with chickens (about 50.6%). Protein interactions analysis showed that there was interaction between sheep CD3 and CD4, CD247 and other proteins. It has been demonstrated that two ζ chains of CD3 molecule were encoded by CD247 gene, which form a TCR/CD3 complex with T cell antigen receptor αβ (TCRαβ) or γδ (Tcrγδ) and CD3γ, ε, δ chains in a non-covalent bond (36). TCR mainly recognizes and binds MHC antigenic peptide complexes, and CD3 transduces the signals recognized by TCR and induce activation of T lymphocyte. The initiation of T lymphocyte activation is determined by the level of TCR/CD3 membrane expression levels (37). Our predictive analysis shows that the molecular weight of sheep CD3 was 21555.57 U, the theoretical isoelectric point (PI) was 6.73, the predicted instability index (PI) was 19.83, and there were more amino acids in the hydrophilic region than in the hydrophobic region. It was indicated that CD3 was an acidic, hydrophilic, and stabile protein without extramembrane region and signal peptide. It mainly plays biological roles in the cell membrane, and the analysis of amino acid fractions reveals that leucine is the most predominant. It also suggests that the sheep CD3 recombinant protein would have a good immunogenicity. Polyclonal antibody prepared in this study had good specificity. The construction of recombinant plasmid. The experimental results also confirmed that through the recombinant plasmid was constructed, prokaryotic expression and preparation, the rabbit polyclonal antibody against sheep CD3 recombinant protein had good specificity. These results will lay a foundation for further investigation of the effects of *M. benedeni* infection on T cells in sheep small intestine.

The intestine maintains the normal digestive, absorptive, and secretory function, it also paly an important mucosal defense functions (38). The intestinal mucosal immune system can be divided into induction sites and effector sites (39). The former mainly contains M cells, dendritic cells (40), macrophages (41) and intestinal epithelial cells (42), which are mainly responsible for the uptake and transport of antigens. The latter includes IELs and LP lymphocytes, where transmitted antigens are activated to produce antibodies and various immune factors (43). IEL, as the first immune cell to interact with foreign antigens, microorganisms and parasites in the body’s immune system (44), can induce local and systemic immune responses to clear antigens by secreting related cytokines (45). The CD3 molecule transduces activation signals generated by T cell receptors to recognize antigens (46). So the determination of the change characteristics of CD3^+^ T lymphocytes is very important to evaluate the intestinal immune response to parasitic infection.

The results of this study showed that in the control group, CD3^+^ T cells were distributed in the mucosal epithelium and the lamina propria around the intestinal gland, and their densities were different, among which the distribution density in LP of the jejunum was higher than that in LP of the duodenum and ileum. Our previous studies have confirmed that under physiological conditions, the distribution of IgA^+^, IgG^+^, and IgM^+^ cells in the small intestine of sheep presents obvious local specificity (47). Therefore, under normal conditions, the distribution characteristics of CD3^+^ T cells in the small intestine of sheep are similar to those of antibody secreting cells, both of which have significant local specificity.

*M. benedeni* infection did not change the spatial distribution of CD3^+^ T cells, but led to increased distribution density of CD3^+^ T cells in each intestinal segment. CD3^+^ T cells were significantly increased in LP and IEL in duodenum and ileum compared with in jejunum. This characteristic change is exactly the opposite of the castration effect of *M. benedeni* infection on intestinal antibody secreting cells (48). The function of IEL can directly affect the integrity of mucosal immune barrier (49). Studies have shown that in a mouse model of *Eimeria vermicularis* infection, the number of IEL increases sharply when the number of coccidium oocysts increases (50). Therefore, the increase in the number of CD3^+^ T cells in IEL can be considered to play an important immunomodulatory and immunoprotective role in *M. benedeni* infection. The LP is the main effect site of mucosal immune response. A large amount of IgA secreted by plasma cells can enter the mucosal surface through the mediation of secretory segment to neutralize antigens (51). After infection, the distribution density of CD3+ T cells in LP increased by 33.57% (duodenum), 25.82% (jejunum) and 27.07% (ileum) respectively. Studies have shown that helminth infection can induce immune regulation of autoimmune-mediated inflammatory diseases, promote Th2 cells balance, facilitate IgE class conversion or activation of polyclonal B cells, and significantly increase IgE expression levels (52). This anti-inflammatory state is thought to be driven by T and B regulatory cells and parasite secretions that have the ability to promote immune regulation. Therefore, the results of this study suggest that after infection with *M. benedeni*, the number of CD3^+^ T cells in each intestinal segment of the host increases, and the cellular immune response is significantly enhanced, which is conducive to mediating the host mucosal immune response by CD3^+^ T cells, maintaining the integrity of the mucosal epithelium, and inhibiting intestinal mucosal hypersensitivity. It plays an important regulatory role in anti-bacterial (53), anti-viral (54), anti-infection and anti-local cell carcinogenesis (55, 56), reducing mechanical damage (57), diluting metabolites (58), and neutralizing toxins (59) produced by worms. This study provides an important basis for understanding the molecular mechanism of parasite infection and revealing the interaction between parasite and host, and lays a foundation for studying the changes of different subtypes of T cells. However, the effect of *M. benedeni* infection on the increase of T lymphocytes in sheep small intestine was significantly different in different intestinal segments, which may be related to the metabolites secreted by *M. benedeni* infection or the cell differentiation of the host mucosal epithelium, which needs to be confirmed by further studies.

## 5 Conclusion

In this study, specific rabbit anti-sheep CD3 polyclonal antibody was successfully prepared. CD3^+^ T cells were dispersed in the LP surrounding intestinal glands and intestinal epithelium of sheep small intestine, and their distribution density was relatively high, especially in IEL, the distribution density in jejunum was higher than that of in duodenum and ileum. The spatial distribution of CD3^+^ T cells in the small intestine of sheep was not changed after infection by *M. benedeni*, but the distribution density of CD3^+^ T cells in each intestinal segment was increased. It is suggested that *M. benedeni* infection leads to a significant increase of CD3^+^ T cells in the small intestine, thereby enhancing cellular immunity or strengthening mucosal immunity against *M. benedeni* infection. In addition, the high distribution density of CD3^+^ T cells in the IEL and LP in all intestinal segments provides the basis for studying mucosal immunity and maintenance epithelial integrity, It also plays a role in inhibiting intestinal mucosal hypersensitivity and the recogniting whether epithelial cells are infected by bacteria and viruses or not. This lays the foundation for further studies on the regulatory mechanisms of the intestinal mucosal immune system against *M. benedeni* infection in sheep.

## Data availability statement

The original contributions presented in the study are included in the article/supplementary material, further inquiries can be directed to the corresponding author.

## Ethics statement

The animal study was approved by Animal Care and Use Committee (IACUC) of College of Veterinary Medicine of Gansu Agricultural University (Approval No.: GSAU-Eth-VMC-2021-021). The study was conducted in accordance with the local legislation and institutional requirements.

## Author contributions

WC: Conceptualization, Methodology, Validation, Writing – original draft, Writing – review & editing. WY: Supervision, Writing – review & editing, Methodology. JP: Methodology, Writing – review & editing. ZH: Writing – review & editing, Validation. BW: Writing – review & editing, Supervision. BX: Writing – review & editing, Investigation. XF: Investigation, Writing – review & editing. WH: Investigation, Writing – review & editing. WW: Writing – review & editing, Project administration. WZ: Project administration, Writing – review & editing, Conceptualization, Funding acquisition, Supervision, Validation.

## Footnotes

^1^ http://www.expasy.ch/swissmod/SWISS-MODEL.html

^2^ https://services.healthtech.dtu.dk/service.php?NetPhos-3.1

^3^ https://services.healthtech.dtu.dk/service.php?NetNGlyc-1.0
